# PDIA3 inhibits mitochondrial respiratory function in brain endothelial cells and *C. elegans* through STAT3 signaling and decreases survival after OGD

**DOI:** 10.1186/s12964-021-00794-z

**Published:** 2021-12-18

**Authors:** Matt. P. Keasey, V. Razskazovskiy, C. Jia, E. D. Peterknecht, P. C. Bradshaw, T. Hagg

**Affiliations:** 1 Department of Biomedical Sciences, Quillen College of Medicine, East Tennessee State University, PO Box 70582, Johnson City, TN 37614 USA; 2 Sandwell and West, Birmingham Hospitals NHS Trust, Birmingham, UK

**Keywords:** PDIA3, Mitochondria, STAT3

## Abstract

**Background:**

Protein disulfide isomerase A3 (PDIA3, also named GRP58, ER-60, ERp57) is conserved across species and mediates protein folding in the endoplasmic reticulum. PDIA3 is, reportedly, a chaperone for STAT3. However, the role of PDIA3 in regulating mitochondrial bioenergetics and STAT3 phosphorylation at serine 727 (S727) has not been described.

**Methods:**

Mitochondrial respiration was compared in immortalized human cerebral microvascular cells (CMEC) wild type or null for PDIA3 and in whole organism *C. Elegans* WT or null for *pdi*-3 (worm homologue). Mitochondrial morphology and cell signaling pathways in PDIA3-/- and WT cells were assessed. PDIA3-/- cells were subjected to oxygen–glucose deprivation (OGD) to determine the effects of PDIA3 on cell survival after injury.

**Results:**

We show that PDIA3 gene deletion using CRISPR-Cas9 in cultured CMECs leads to an increase in mitochondrial bioenergetic function. In *C. elegan*s, gene deletion or RNAi knockdown of *pdi-3* also increased respiratory rates, confirming a conserved role for this gene in regulating mitochondrial bioenergetics. The PDIA3-/- bioenergetic phenotype was reversed by overexpression of WT PDIA3 in cultured PDIA3-/- CMECs. PDIA3-/- and siRNA knockdown caused an increase in phosphorylation of the S727 residue of STAT3, which is known to promote mitochondrial bioenergetic function. Increased respiration in PDIA3-/- CMECs was reversed by a STAT3 inhibitor. In PDIA3-/- CMECs, mitochondrial membrane potential and reactive oxygen species production, but not mitochondrial mass, was increased, suggesting an increased mitochondrial bioenergetic capacity. Finally, PDIA3-/- CMECs were more resistant to oxygen–glucose deprivation, while STAT3 inhibition reduced the protective effect.

**Conclusions:**

We have discovered a novel role for PDIA3 in suppressing mitochondrial bioenergetic function by inhibiting STAT3 S727 phosphorylation.

**Supplementary Information:**

The online version contains supplementary material available at 10.1186/s12964-021-00794-z.

## Background

PDIA3 is a protein disulfide isomerase that promotes protein folding in the ER [1–4]. PDIA3 is also present in the cytoplasm, nucleus, mitochondria and plasma membrane caveolae [5–7], where it mediates glucose stress responses [8] and modulates transcriptional regulation [8]. Human PDIA3 has homology with proteins in distant eukaryotes*,* with approximately 50% primary sequence homology with PDI-3 of *C. elegans*, suggesting vital conserved roles between species. PDIA3 deletion in mice is lethal at E13.5 [9] and deletion of *pdi-3* in *C. elegans* causes morphological defects (“dumpy”) and dysregulated collagen deposition [10]. In humans, PDIA3 is ubiquitously expressed and has been implicated in pathology such as cancer progression [11], Huntington’s disease [12], amyotrophic lateral sclerosis [13], and traumatic brain injury [14]. Mitochondrial dysfunction is a hallmark of these conditions [15–18], but little is known about PDIA3’s role in mitochondrial homeostasis and function.

Within mitochondria, PDIA3 promotes the intrinsic apoptotic pathway and mitochondrial permeabilization [6, 19, 20]. Furthermore, PDIA3 can increase mitochondrial calcium uptake by transcriptionally upregulating the mitochondrial calcium uniporter [21]. Finally, PDIA3 is present at ER-mitochondrial contact sites, suggesting a possible role for non-transcriptional regulation between these organelles [22]. A general protein disulfide isomerase inhibitor can protect against reductions in ATP levels in an in vitro Huntington disease model [12], supporting the notion that PDIA3 might inhibit mitochondrial bioenergetic function.

A potential mechanism through which PDIA3 might modulate mitochondrial bioenergetics is through its interaction with STAT3. PDIA3 is a chaperone for STAT3 and can modulate STAT3 transcriptional activity by inhibiting or promoting phosphorylation of the Y705 residue [8, 9, 23–25] and inhibiting STAT3 S727 phosphorylation [23, 26]. Phosphorylation of only the S727 residue targets STAT3 for mitochondrial import [27], where it promotes bioenergetic function and reduces reactive oxygen species (ROS) [28, 29]. In cardiomyocytes, STAT3 prevents mitochondrial permeabilization through inhibiting mitochondrial permeability transition pore opening and reduces toxic ROS levels during ischemia/reperfusion injury [30, 31]. Phosphorylated S727 (pS727) STAT3 also preserves mitochondrial function during post-ischemic conditioning after injury [32]. We have shown that reduced mitochondrial pS727 STAT3 in endothelial cells is associated with ER stress-induced cell death [33], while increased mitochondrial pS727 STAT3 improves bioenergetic function and endothelial cell survival [34]. Brain endothelial cells have very high concentrations of mitochondria [35, 36], which are important for maintaining the blood–brain-barrier [37, 38]. Here, we investigated whether PDIA3 suppresses mitochondrial bioenergetic function through STAT3 by using CMECs, a well-differentiated human brain endothelial cell line. We used *C. elegans* to test the role of the homologue, PDI3, in an intact animal because the PDIA3 knockout in mice is embyronically lethal [9]. Additionally, we tested whether PDIA3 deletion would be protective against OGD injury in cultured CMECs.

## Methods

### CMECs and *C. elegans*

CMECs are an immortalized clone of well-differentiated microvascular endothelial cells originally obtained from brain tissue of a female epilepsy patient [39, 40] and were expanded and maintained at 37 °C and 5% CO_2_ as described [41]. We chose to use CMEC’s as these cells are highly transfectable which allows for genetic manipulation and brain endothelial cells have robust expression of PDIA3 [42]. N2 and TP66 *C. elegans* strains were obtained from the Caenorhabditis Genetics Center (CGC). *C. elegans* were chosen as a model because a knockout is available (TP66) while PDIA3 KO in mice leads to a lethal phenotype [9]. TP66 worms have a *pdi-3* gene deletion, originally identified by genetic screens of mutants generated by random UV/trimethylpsoralen and were backcrossed four times [10]. TP66 worms were found to be homozygous for the *pdi-3* deletion with genotyping performed by PCR of isolated genomic DNA (primers: 5′-CGTGTCTTGAAAGTTGCTC-3′, 3′-CCCTCTAACTTCGAACATTGG-5′). No difference was observed between the strains in the developmental time from L1 larvae to adulthood. Worms were grown on Nematode Growth Media agar plates with a surface coating of live *OP50 E. coli* feeder bacteria [43]. The worms were synchronized by bleaching followed by overnight suspension in M9 buffer to obtain L1 stage larva and experiments were performed at the L3/L4 stage.

### Bioenergetic measurements

Oxygen consumption rate (OCR) assays were performed as described [44] with minor modifications. Briefly, we used an XF HS mini-Seahorse analyzer (Agilent) with Flux Packs (103,022–100, Agilent). For quantification of bioenergetic functions, basal respiration was calculated as baseline OCR minus OCR level after antimycin A, ATP-linked respiration as the level after baseline minus oligomycin, reserve (spare) capacity as the level after FCCP minus baseline, and maximal respiration as the OCR level reached after FCCP minus the antimycin A level. CMECs were plated onto collagen type 1 (3440–005-01, Cultrex, 150 µg/ml in H_2_O) at ~ 20,000 cells/well. L3/4 stage worms were fed heat-killed OP50 *E. coli* bacteria for 48 h to avoid background respiration from *E. coli* that may have stuck to *C. elegans* after washing. Bacteria were heat killed by two cycles of flash freezing in liquid nitrogen followed by heating to 70 °C for 2 h. Immediately prior to analysis, worms were washed 5× with 0.1 M NaCl to remove excess bacteria before counting and plating ~ 75 worms/well in M9 medium. For worms, 40 mM sodium azide was used instead of antimycin A and oligomycin was excluded because it cannot readily diffuse through the worm cuticle. *C. elegans* were counted by an investigator who was blinded to the genotype and treatment of the worms from images taken after the assays were performed.

### Fluorometric and morphological measurements

Fluorometric measurements were carried out as described [34]. CMECs were plated at 43 cells/mm^2^ (~ 20,000 cells/well) for 48 h on type 1 rat collagen, then incubated with 1 µg/ml Hoechst 33,342 (H1399, Fisher) to stain the nuclei, 0.5 µM MitoTracker Red CMXRos (M7512, Invitrogen) to assess mitochondrial membrane potential, 5 µM MitoSox Red (M36008, Thermo) for superoxide measurements or 0.2 µM LysoTracker Red (L7528, Thermo) to assess lysosomal mass. Mitochondrial surface area and volume were measured using IMARIS software [45] using CMECs after incubation with MitoTracker Green (M7514, Invitrogen) and imaging by confocal microscopy (20–30 image planes in the Z-axis, Leica TCS SP8).

### Western protein detection

Western blots were performed with primary antibodies (Table 1) as described [46]. Quanititative capillary western (WES) for small amounts of mitochondrial protein were performed as described [41]. Lysates were loaded at 1 μg per capillary into plates (SM-W002, ProteinSimple) [41]. Detection was performed by chemiluminescence (DM-001, ProteinSimple) and data analysis was performed using Compass software (Version 4.0.0).

**Table 1:** Primary antibodies for western blotting

Protein	Cat.#	Research resource identifier (RRID)	Dilution
α-tubulin	2125	AB_2619646	1:2,000
β actin	4970	AB_2223172	1:5,000
FAK	3285	AB_2269034	1:1,000
FAK pY397	3283	AB_2173659	1:1,000
GAPDH	5174	AB_10622025	1:5,000
PDHA1	3205	AB_2162926	1:1,000
PDIA3	2881	AB_2160840	1:1,000
STAT3	12640	AB_2629499	1:1,000
STAT3 pS727	9134	AB_331589	1:1,000
STAT3 pY705	9145	AB_2491009	1:1,000

All antibodies were from Cell Signaling Technologies

### CRISPR-Cas9-mediated PDIA3 deletion

To create permanent PDIA3-/- CMECs, we used CRISPR-Cas9 as described [34]. We chose to perform genetic ablation to enable specific knockdown of targeted genes. Pharmacological inhibition of PDIA3 is difficult because available PDI inhibitors lack specificity [47, 48]. Cas9 guide RNAs were designed using CHOPCHOP [49] targeting exon 4 of the human PDIA3 gene. Oligonucleotides (5′-GATCGTGTCAGCCACTTGAAGAAGC-3′, 3′-AAAACGCTTCTTCAAGTGGCTGACA-5′) were annealed and directionally cloned with BamHI and BsmBI into a pCas-Guide plasmid (Ge100002, Origene) encoding Cas9 downstream of a CMV promoter. Genotyping was performed by standard PCR using primers flanking the predicted cut site (5′-GATCGGGTAACGAGTTGGTCCCGC-3′, 3′-AAAACGTAGGCCCAACAGTTCCCC-5′).

### siRNA and PDIA3 transfections

CMECs were transfected with an siRNA targeting PDIA3 (L-003674, Horizon) or a non-targeting control siRNA (D-001810–10, Horizon), using 0.25% [v/v] Lipofectamine 3000 and 25 nM siRNA, as described [41]. Cells were maintained for 6 days before protein isolation. PDIA3 overexpression was performed by transfection of mammalian expression cassettes (pcDNA 3.1 + , V79020, Thermo) 72 h before OCR measurements. First, PDIA3 was cloned by PCR-amplification using cDNA as template material, reverse transcribed (Cat# 18,091,050, Invitrogen) from purified CMEC RNA. Amplified product (5′-CGCGGATCCCACCTCGCCGCCATGCGC-3′, 3′-CGGGAATTCTTAGAGATCCTCCTGTGCCTTCTTC-5′) was directionally cloned into the pcDNA 3.1 ( +) backbone downstream of a CMV promotor, using BamHI and EcoRI sites at the 5′ and 3′ ends, respectively and confirmed by Sanger Sequencing. Transfections of plasmid DNA isolated from endotoxin free midi-prep kits (D6915, Omega Biotek) were performed as described [34], with 0.5 µg DNA/well in Seahorse plates. Control cells were transfected with green fluorescent protein (GFP) constructs. For siRNA knockdown of PDI-3, N2 WT strain worms were fed HT115(DE3) bacteria expressing an siRNA construct targeting PDI-3 (RCE1182-202297109, Clone: H06O01.1, Horizon Discovery) up to L3/4 or a control empty vector, in the presence of ampicillin (100 μg/ml), tetracycline (10 μg/ml) and IPTG (Isopropyl β-D-1-thiogalactopyranoside, 1 mM) to induce RNAi expression.

### RT-qPCR

RT-qPCR was performed using Taqman probes as described [46]. Analyses were performed using the delta-delta-Ct method with relative gene expression first normalized to GAPDH mRNA and then expressed relative to the experimental controls. Primers for human PDIA3 (Hs04194196_g1) and GAPDH (Hs02786624_g1) or mouse PDIA3 (Mm00433130_m1) and GAPDH (Mm99999915_g1) were from ThermoFisher and designed to cross exon borders to specifically target cDNA.

### OGD

OGD was performed as described [50]. CMECs were plated at 200,000 cells/well and maintained for 48 h before a 24 h incubation in glucose-free deoxygenated DMEM in a hypoxia chamber (5% CO_2_ in 95% nitrogen). Afterwards, Hoechst was added to the medium and CMECs were imaged with a 20 × objective for quantification with ImageJ software, using a grid set to 100,000-pixel size, with nine squares per image counted blindly. Live cells were characterized by large diffuse nuclei and were counted blindly. Lactate dehydrogenase release was used as an indicator of cell death and measured according to manufacturer’s protocol (786-210, G-Biosciences).

### Statistics

Sample sizes were based upon power analysis (G*power) and indicate independent experiments in the results. Statistical analyses used GraphPad Prizm with Student’s t-test for two groups, one-way ANOVA for more than two groups, and a two-way ANOVA for multiple variables in more than two groups with normally (gaussian) distributed data.

## Results

### PDIA3 inhibits mitochondrial bioenergetic function in CMECs

**Fig. 1:**
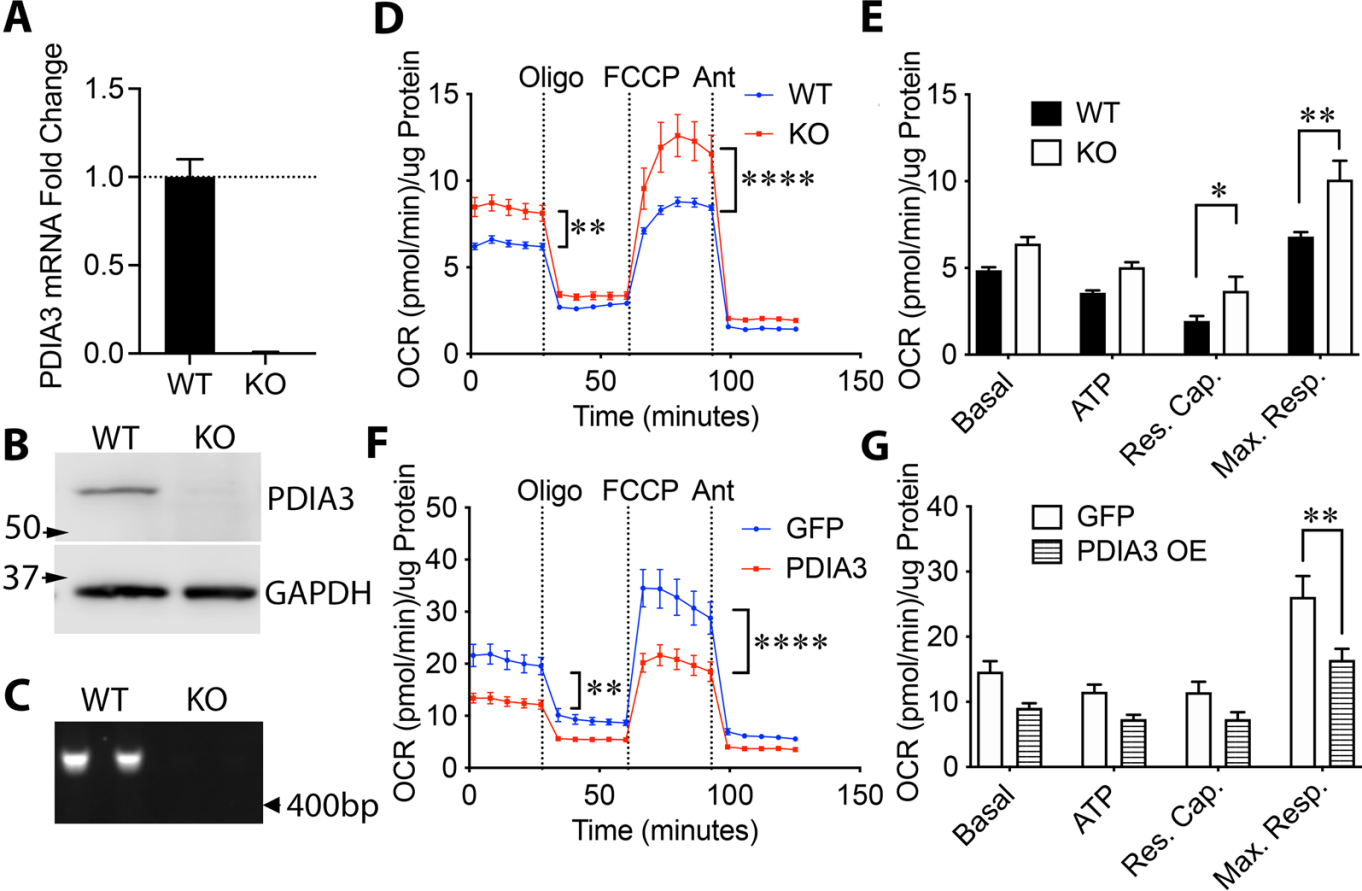
PDIA3 inhibits mitochondrial respiratory function in cultured human brain endothelial cells. **a** PDIA3 gene deletion or PDIA3-/- (KO) was achieved in immortalized hCMEC/D3 cells (CMECs) by CRISPR-Cas9 and confirmed by RT-qPCR (delta–delta-Ct normalized first to GAPDH) and western blotting (**b**). WT CMECs were transfected with non-targeting gRNA. **c** Genotype was further confirmed by PCR from genomic CMEC DNA surrounding the target cut site. **d** Respiratory rates, as measured by oxygen consumption rate (OCR) by Seahorse analysis, were increased in PDIA3-/- CMECs relative to WT cells (repeated measures ANOVA. n = 3 per condition). **e** Reserve (spare) capacity (Res. Cap.) and maximal respiratory capacity (Max. Resp.) were increased but not basal respiratory rate and ATP-linked respiration (n = 3 each, two-way ANOVA). **f** The PDIA3-/- phenotype of increased bioenergetic function was largely reversed by overexpressing WT human PDIA3 compared to GFP control transfection (**g**)

PDIA3 is known to regulate STAT3 signaling but its role in bioenergetic function is unknown. Genetic deletion of PDIA3 in CMECs with CRISPR-Cas9 was confirmed by qPCR (Fig. 1a, n = 3), western blotting (Fig. 1b) and genomic PCR with primers flanking the targeted Cas9 cut site (Fig. 1c). WT control CMECs were transfected with Cas9 and a scrambled gRNA. PDIA3-/- CMECs had increased oxygen consumption rates (OCRs) (Fig. 1d) with an increase in reserve capacity (Fig. 1e, n = 3, *p* < 0.01), indicating a greater spare ATP-generating capacity and an increased ability to respond to stress or higher energy demand [51]. The maximum respiratory capacity was also increased in PDIA3-/- relative to control-transfected WT cells (Fig. 1e, n = 3, *p* < 0.01), indicating these cells have increased maximum respiratory rates. As a confirmation, PDIA3 overexpressed in PDIA3-/- CMECs suppressed bioenergetic function (Fig. 1f, n = 3, *p* < 0.001), particularly maximal respiratory capacity (Fig. 1g, n = 3, *p* < 0.01) relative to GFP-transfected controls.

### PDI-3 inhibits bioenergetic function in *C. elegans*

**Fig. 2:**
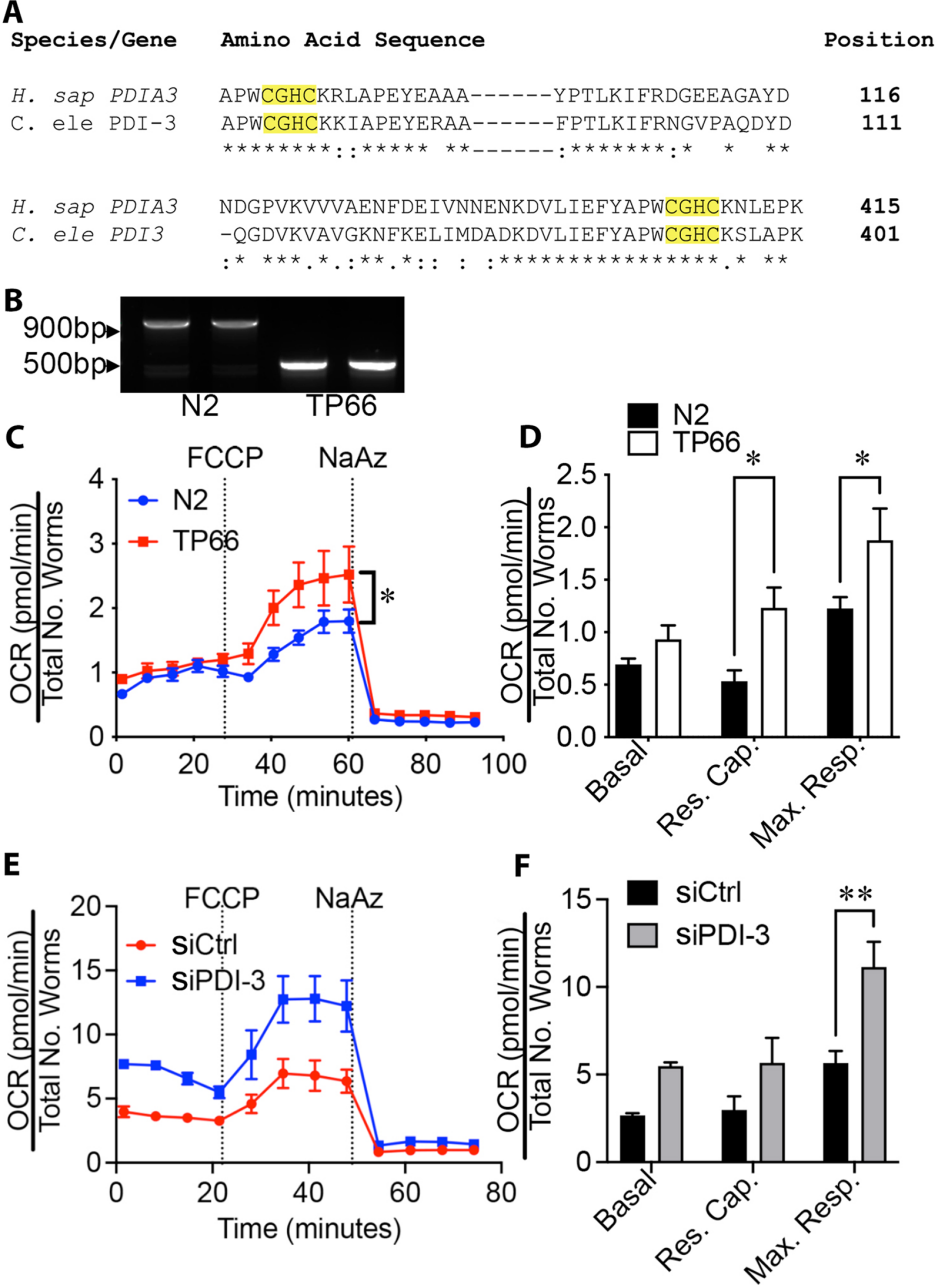
PDI-3, a PDIA3 ortholog, inhibits respiratory function in whole *C. elegans* worms. **a** PDIA3 is highly conserved in the eukaryotic genome, having approximately 50% amino acid sequence homology between humans and worms (thioredoxin domains highlighted in yellow). **b** The PDI-3 gene deleted TP66 strain *C. elegans* genotype was confirmed by PCR targeting genomic DNA surrounding the excision site. **c, d PDI-3** is deficient in the TP66 strain of *C. elegans* and leads to an increase in reserve capacity and maximal respiration compared to N2 WT worms after treatment with the mitochondrial oxidative phosphorylation uncoupler FCCP (with ~ 75 worms from n = 6 plates, two-way ANOVA, *p* < 0.05). **e–f** Acute knockdown of PDI-3 by siRNA in N2 strain worms increased maximal respiration (n = 3, two-way ANOVA, *p* < 0.05) relative to control (empty vector) fed worms

We determined whether PDIA3 inhibited mitochondrial function in an evolutionarily distant species and in an intact multicellular organism. The *pdi-3* gene is the *C. elegans* worm ortholog of mammalian PDIA3 and has ~ 50% amino acid homology and conserved catalytic thioredoxin domains (Fig. 2a). TP66 worms have a *pdi-3* deletion [10] as confirmed by PCR showing a shorter amplicon due to a lesion around exon 3 [10] (Fig. 2b). OCR was increased in TP66 relative to WT N2 strain worms (Fig. 2c), showing increased reserve capacity and maximal respiration (Fig. 2d, n = 6 independent plates, *p* < 0.05). Moreover, RNAi knockdown of *pdi-3* in WT N2 worms increased OCR, particularly maximal respiration, relative to control-transfected N2 worms (Fig. 2e).

### PDIA3 reduces mitochondrial activity

**Fig. 3:**
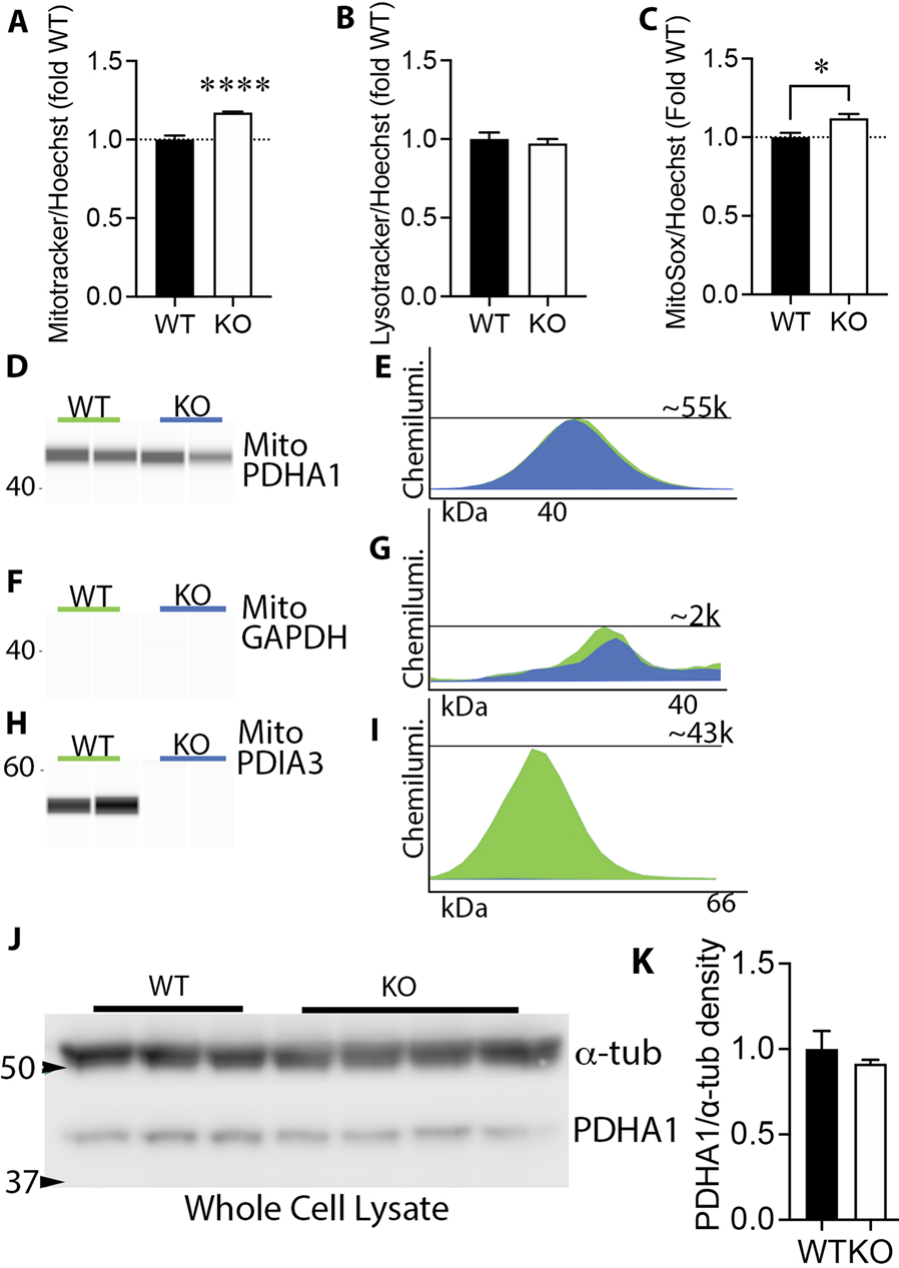
Mitochondrial membrane potential and ROS, but not mitochondrial mass, is increased in CMECs. **a** MitoTracker Red CMXRos dye is a potentiometric dye that accumulates in active mitochondria. Fluorometry showed that MitoTracker Red CMXRos intensity was greater in PDIA3-/- CMECs relative to WT (~ 20%, *p* < 0.001, n = 4, Student’s t-test). Values were normalized to nuclear Hoechst dye to account for potential variations in cell number and expressed as a fold of WT. **b** The fluorescence of LysoTracker Red, a dye that accumulates in lysosomes, was not different between genotypes. **c** Superoxide was measured using MitoSox Red. PDIA3-/- CMECs had higher levels of superoxide (n = 4, *p* < 0.01) relative to WT CMECs. **d** Synthetic bands derived from peak chemiluminescent intensity of **e** capillary western blotting of proteins from isolated mitochondria show that the amount of pyruvate dehydrogenase (PDHA1) was not different between PDIA3-/- and WT CMECs. The chemiluminescence peak amount is indicated by the number 55 K indicating 55,000. **f, g** GAPDH, a cytoplasmic protein, was largely absent, indicating the purity of the mitochondrial sample. **h, i** PDIA3 was present in mitochondrial fractions of WT, but not PDIA3-/-, CMECs. **j, k** Regular western blots of whole cell lysates show that the PDIA3-/- and WT CMECs have similar amounts of the mitochondrial protein PDHA1, indicating a similar mitochondrial mass. The cytoplasmic household protein α-tubulin was used as loading control

MitoTracker Red CMXRos dye accumulation depends on mitochondrial membrane potential [52]. PDIA3-/- CMECs treated with this dye had greater red fluorescence than WT CMECs (Fig. 3a, n = 6, *p* < 0.001) when normalized to nuclear Hoechst stain to account for variability in cell numbers [33]. In contrast, the signal from LysoTracker Red dye, which fluoresces when localized to acidic organelles such as lysosomes and autophagosomes, was not different between genotypes (Fig. 3b, n = 6), suggesting that PDIA3-/- CMECs did not have alterations in lysosomal or autophagosomal mass or mitophagy [53]. PDIA3-/- cells had increased MitoSox Red fluorescence, indicating increased mitochondrial superoxide production (Fig. 3c, n = 4, *p* < 0.01), likely due to the increased mitochondrial electron transport chain activity as indicated by the by the increased OCRs.

### PDIA3-/- CMECs have normal mitochondrial mass

**Fig. 4:**
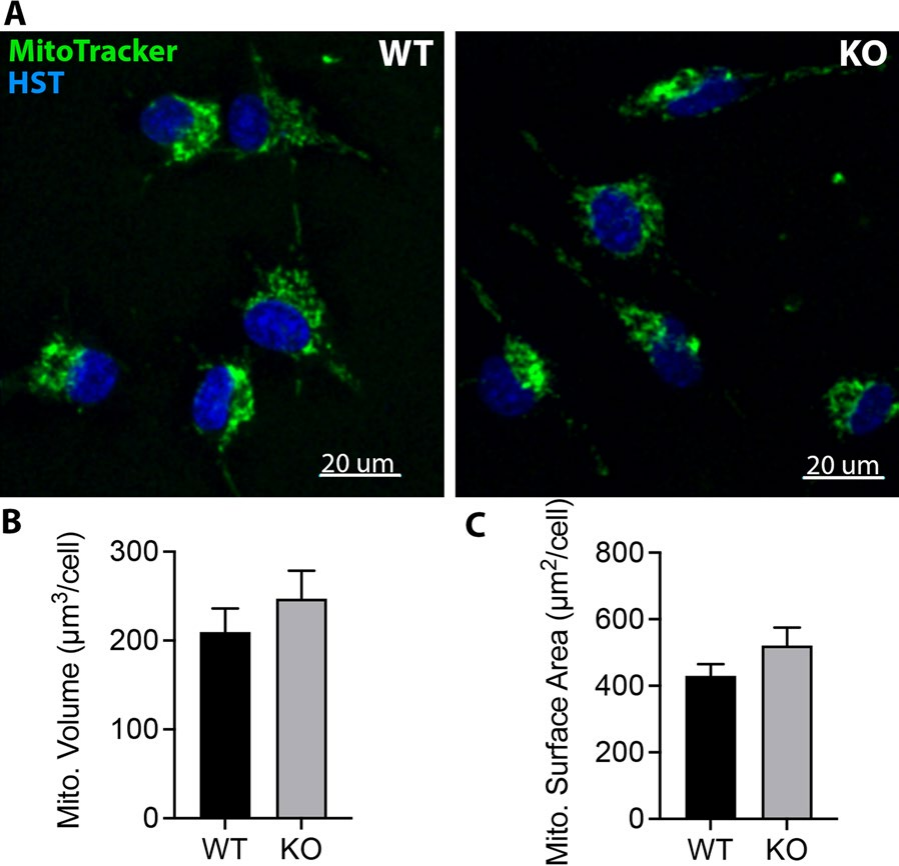
Mitochondrial surface area or volume is not altered in PDIA3-/- CMECs. **a** WT or PDIA3-/- (KO) CMECs were stained with MitoTracker Green dye and imaged by confocal microscopy. A total of 32 cells were imaged across 3 independent experiments and mitochondrial surface area and volume were analyzed using IMARIS software. Neither total mitochondrial surface area (**b**) or volume (**c)** were different between WT and PDIA3-/- CMECs

To exclude the possibility that bioenergetic increases were caused by changes in mitochondrial mass, protein from purified mitochondria were quantified using capillary western analysis. Pyruvate dehydrogenase subunit A1 (PDHA1) is essential for metabolizing the end product of glycolysis into the mitochondrial citric acid cycle and altered PDH activity affects OCR [54]. PDHA1 expression was not different between PDIA3-/- and WT CMECs (Fig. 3d, e). Cytosolic GAPDH was not detected, confirming the purity of the mitochondrial fractions (Fig. 3f, g). PDIA3 was present in WT mitochondrial extracts, but not those from PDIA3-/- CMECs (Fig. 3h, i), confirming PDIA3 is present in the mitochondria. Finally, PDHA1 was equally abundant in whole cell lysates of both genotypes, further suggesting that mitochondrial mass is not altered by PDIA3 knockout (Fig. 3j, k). Moreover, the mitochondrial tubular networks of MitoTracker Green-treated cells appeared similar in PDIA3-/- and WT CMECs, and their estimated mitochondrial surface area (Fig. 4b) and volume visualized by by confocal microscopy were not different (Fig. 4c).

### PDIA3 reduces bioenergetic function through increased STAT3 S727 phosphorylation

**Fig. 5:**
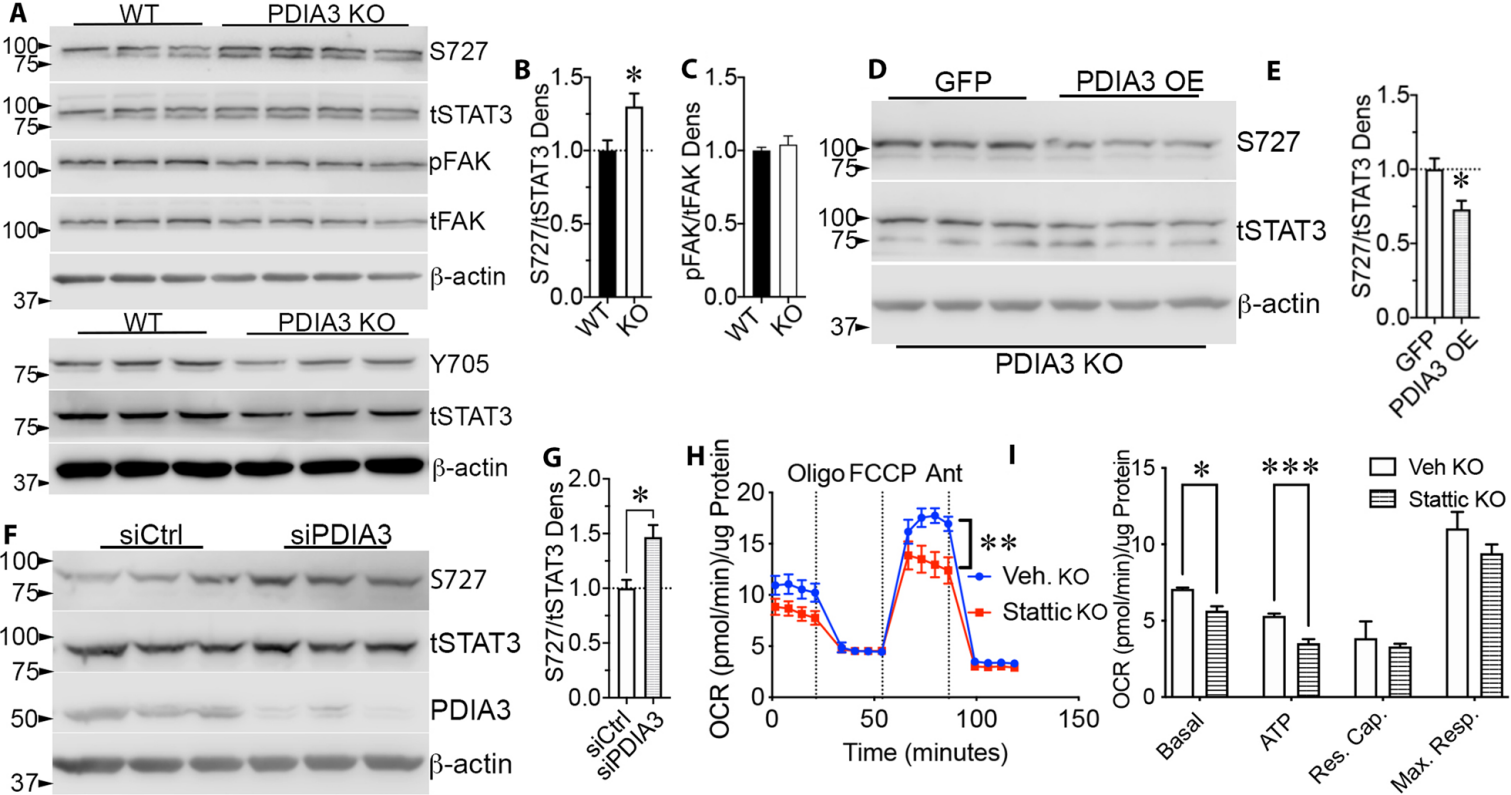
PDIA3 inhibits mitochondrial function by suppressing pS727 STAT3. **a** STAT3 S727 phosphorylation was increased in PDIA3-/- CMECs, as confirmed by densitometry (**b**), * *p* < 0.05, Student’s t-test). Phosphorylation of STAT3 Y705 was not obviously different in PDIA3-/- CMECs, considering the total amounts of STAT3 and the β-actin loading control. We previously found that FAK drives mitochondrial bioenergetics via STAT3 S727 phosphorylation [34] but pFAK was not different in PDIA3-/- (KO) CMECs, as confirmed by densitometry (**c**). **d** Overexpression of WT human PDIA3 in PDIA3-/- CMECs reduced pSTAT3 S727, as seen by western blotting and densitometry (**e**). **f**) The inhibitory effect of PDIA3 on STAT3 S727 phosphorylation was confirmed by siRNA mediated knockdown of PDIA3 (siPDIA3) in WT CMECs relative to a non-targeting control siRNA (siCtrl). Knockdown increased STAT3 S727 phosphorylation (**g**). **h** Finally, we tested whether increased STAT3 S727 phosphorylation was necessary for PDIA3-/- increased respiratory function. Inhibition of STAT3 with stattic in PDIA3-/- CMECs, reduced respiratory function compared within 1 h, as measured by Seahorse assay (n = 6, *p* < 0.01, one-way ANOVA). **i** Quantification showed decreased basal and ATP-linked respiration (n = 6, *p* < 0.01, two-way ANOVA), which indicates PDIA3 suppresses mitochondrial function through inhibition of STAT3

STAT3 S727 phosphorylation increases mitochondrial respiration [55]. PDIA3 binds to STAT3 [9] to modulate Y705 phosphorylation [8, 23, 56] and can also inhibit S727 phosphorylation [23, 26]. Here, PDIA3-/- CMECs showed increased levels of STAT3 S727 phosphorylation (Fig. 5a, b; n = 3 for WT and n = 4 PDIA3-/-, *p* < 0.05). Levels of STAT3 Y705 did not appear to be altered in PDIA3-/- CMECs relative to WT (Fig. 5a). We have shown that integrin-FAK signaling promotes mitochondrial function through STAT3 S727 phosphorylation [34]. However, phosphorylation of FAK Y397 was not decreased in PDIA3-/- CMECs (Fig. 5a, c). The increase in STAT3 pS727 in PDIA3-/- CMECs was reversed by overexpression of PDIA3 compared to control GFP (Fig. 5d, e). Conversely, PDIA3 siRNA reduced PDIA3 protein expression and increased STAT3 pS727 (Fig. 5f, g). We next determined whether PDIA3-/- increased bioenergetic function through STAT3 signaling by using the STAT3 inhibitor, stattic [57], which suppresses STAT3 pS727 and bioenergetic function in bEnd5 endothelial cells [34]. Stattic reduced OCR in PDIA3-/- CMECs (Fig. 5h), with the cells showing lower basal and ATP-linked respiration rates (Fig. 5i, *p* < 0.05, n = 3).

### PDIA3 promotes cell growth

Mitochondrial function is often linked to proliferation and survival [58], which we controlled for as a potential confounding factor in our analyses. PDIA3-/- CMECs grew more slowly between 48 and 72 h after plating (Additional file 1: Fig. S1A, B, *p* < 0.05, n = 3). Release of lactate dehydrogenase, a measure for cell death, was not different between genotypes between 48 and 72 h (Additional file 1: Fig. S1C). Thus, PDIA3 might promote proliferation, but not cell death, under normal conditions. The late differences in proliferation between PDIA3-/- and WT CMECs should not confound the OCR measurements made 24 h after plating or compared within the same genotype. Moreover, the OCR data were normalized to protein content.

### PDIA3 reduces resistance to OGD by inhibiting pS727 STAT3

**Fig. 6:**
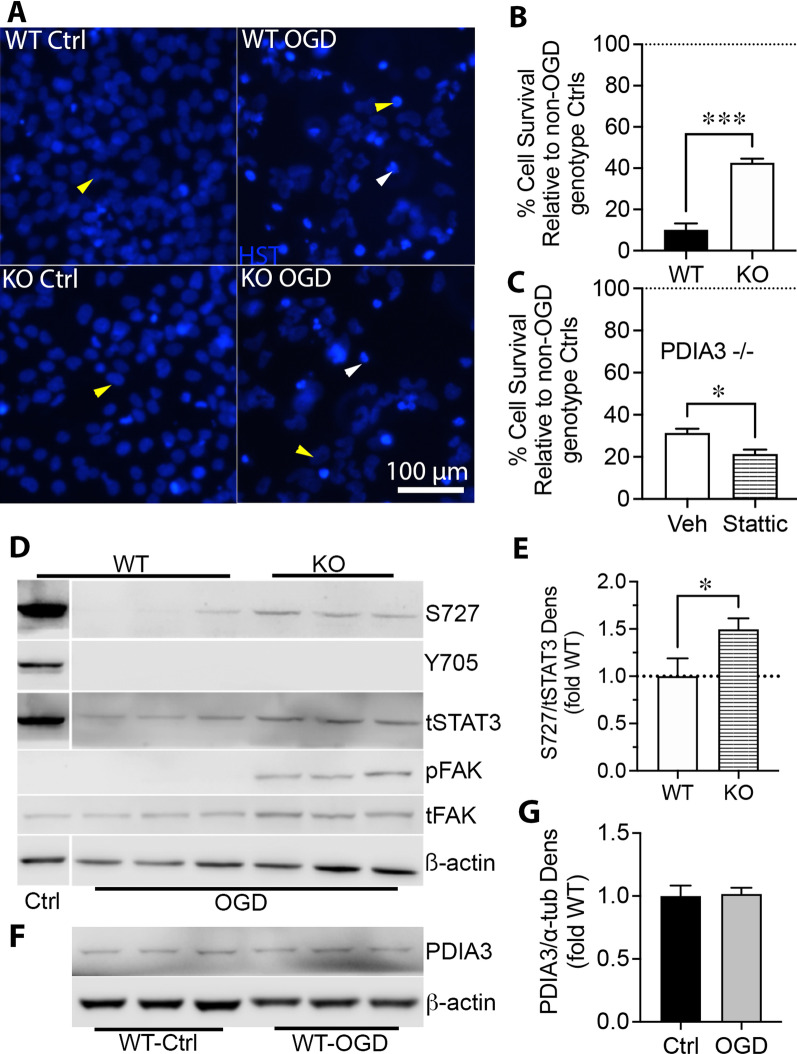
PDIA3 contributes to cell death from oxygen–glucose deprivation. **a** A 24 h period of OGD caused a loss of live (yellow arrows) and appearance of apoptotic (white arrowheads) CMECs compared to normoxic control conditions, as revealed by blue nuclear Hoechst staining. **b** Cell counts from 3 fields/well show that the number of live PDIA3-/- cells was four times higher than WT cells after OGD (*p* < 0.001, n = 3, Student’s t-test). **c** Pharmacological inhibition of STAT3 with stattic reduced the protective effect of PDIA3-/- against OGD in CMECs (Student’s t-test, n = 3, *p* < 0.05). **d** Western blotting shows that PDIA3-/- CMECs had more phosphorylated STAT3 S727, as measured by densitometry (**e**) as well as increased pFAK Y397 which was completely absent in control conditions or WT CMECs undergoing OGD. STAT3 Y705 phosphorylation was lost after OGD in both PDIA3-/- and WT CMECs, indicating Y705 plays no part in PDIA3-/- cell survival. Beta actin was used as a loading control to account for differences in cell survival and STAT3 and actin blots were run on the same gel. **f** PDIA3 expression was not different in WT control (Normoxia) relative to WT OGD CMECs as measured by densitometry (**g**)

PDIA3 can promote cell death by stabilizing mitochondrial calpains and activating apoptosis inducing factor [6]. To test whether PDIA3 alters cell survival under conditions which mimic key aspects of ischemia, we performed OGD, which causes cell death in part by inhibiting mitochondrial bioenergetic function [59]. The role of PDIA3 in OGD is unclear because its expression increased in response to glucose depletion [1], but decreased upon oxygen depletion [60]. PDIA3-/- CMECs had less cell death than WT CMECs after OGD (Fig. 6a, n = 3, *p* < 0.001), with approximately 40% survival in PDIA3-/- and 10% survival in WT CMECs after 24 h of OGD (Fig. 6b, *p* < 0.001, n = 3). Inhibition of STAT3 abolished the protective effect of PDIA3 gene deletion (Fig. 6c). PDIA3-/- CMECs had better preserved pS727 and total STAT3 than WT CMECs after a 24 h OGD (Fig. 6d, e). STAT3 pY705 was completely abolished by OGD treatment in both genotypes (Fig. 6d). Upstream FAK was strongly phosphorylated in PDIA3-/-, but not WT, CMECs following OGD (Fig. 6d). Finally, PDIA3 expression was not altered in WT control CMECs relative to OGD (Fig. 6f–g). These data suggest that PDIA3 decreases survival of CMECs in vitro under OGD conditions through STAT3 inhibition.

## Discussion

Our most important and novel findings are that PDIA3 inhibits mitochondrial respiratory function, S727 STAT3 phosphorylation and cell survival under OGD conditions. The novel role of PDIA3 in downregulating mitochondrial bioenergetics is consistent with proteomics data indicating its potential involvement in modulating aerobic capacity in skeletal muscle [26]. PDIA3 had been shown to inhibit STAT3 S727 phosphorylation and our genetic and pharmacological results demonstrate that this downregulates mitochondrial respiratory function and cell survival under OGD conditions [23]. STAT3 regulation of mitochondrial respiration is dependent upon S727 phosphorylation and subsequent interaction with retinoid-interferon mortality-19 (GRIM19), which mediates mitochondrial import and integration with complex I of the electron transport chain [27, 55]. Thus, loss of PDIA3 and consequently increased pS727 may lead to increased availability of STAT3 for mitochondrial import. Although PDIA3 binds to STAT3 by direct protein–protein interaction [23] and modulates its signaling [61, 62], the mechanism by which it suppresses pS727 is unclear, as PDIA3 is not a phosphatase. PDIA3 is known to chaperone proteins [7] and it might act as a chaperone for serine phosphatases that dephosphorylate S727 STAT3 and bring them in close proximity to STAT3 S727. PDIA3-STAT3 association typically occurs in either the cytosol [63] or the nucleus [8]. However, like others [20], we found PDIA3 within mitochondria as well, suggesting that PDIA3 could promote dephosphorylation of pS727 within mitochondria. We have shown that STAT3 S727 is dephosphorylated in isolated mitochondria, indicating the presence of mitochondrial-resident serine phosphatases that can perform this function [34]. It remains to be determined whether PDIA3 exerts its observed influence on mitochondrial respiration mostly from its binding to complex I on the inner membrane of the organelle or indirectly from elsewhere.

PDIA3 might also contribute to cell survival and death through other pathways that affect mitochondria. Depletion of the related PDIA1 in endothelial cells causes increased mitochondrial fission and ROS production, leading to cellular senescence [64], although bioenergetic function was not tested. Mitochondrial fragmentation and increases in ROS have been observed in hyperglycemic conditions [65]. A potential role of PDIA3 in decreasing mitophagy is suggested by its role in autophagy in pancreatic beta cells leading to apoptosis [66]. Here, PDIA3 deletion did not affect mitochondrial mass or morphology, but, like depletion of PDIA1, it increased ROS production. Thus, increased bioenergetics upon PDIA3 deletion in CMECs and *C. elegans* is likely the result of increases in the activity of key electron transport chain complexes. Vitamin D restores autolysosomal function in gut epithelial cells through activation of PDIA3 [24]. Although changes in mitophagy and mitochondrial turnover could result in altered bioenergetic capacity, we did not detect differences in lysosomal content between WT and PDIA3-/- CMECs. On the other hand, PDIA1, like PDIA3, contains the prototypical -CGHC- thioredoxin amino acid sequence at its amino and carboxyl termini, which can reduce ROS accumulation. Loss of either one may reduce ROS scavenging capacity within a cell. In our study, PDIA3-/- CMECs showed an increased mitochondrial membrane potential, as well as greater ROS production. This is consistent with a role for PDIA3 as an inhibitor of bioenergetic capacity.

Our study also suggests that PDIA3 inhibition of mitochondrial function is not restricted to mammalian cells. In *C. elegans,* a homolog of mammalian STAT proteins STA-1 has only 30% homology to STAT3 and no data exists on whether PDI-3 interacts with STA-1 [67]. The shortened (dumpy) morphological phenotype of *pdi-3* deleted worms [10] is likely caused by decreased collagen crosslinking and extracellular matrix stabilization due to the lack of PDI-3 transglutaminase activity, as well as the loss of chaperone function in the ER. However, the dumpy phenotype may be enhanced by the increased respiration which produces increased amounts of ROS that are known to modulate cuticle remodeling during molting [68, 69]. PDIA3 may also play a role in suppressing muscle cell functions in which PDIA3 has a potential involvement in aerobic capacity [26]. PDIA3 expression is increased in the gastrocnemius muscle of 30 month old relative to younger 7 month old rats [70] and in chronological muscle ageing in post-menopausal women [71], potentially explaining in part why aged skeletal muscle has reduced oxidative capacity relative to young muscle [72].

Our data also suggest that PDIA3 plays a detrimental role under pathological conditions such as OGD, which mimics key aspects of ischemia. Importantly, both OGD and ischemia are characterized by mitochondrial bioenergetic failure [33]. Long-term bioenergetic dysfunction leads to apoptosis [73]. PDIA3 protein expression is upregulated after OGD in SHSY-5Y neuronal cells [74], but was not altered in the CMECs. Mitochondria play a key role in apoptosis [73], releasing cytochrome C through the mitochondrial permeability transition pore, which STAT3 can suppress [75]. PDIA3 is known to stabilize expression of mitochondrial calpains and cleavage of apoptosis inducing factor, thereby contributing to increased apoptosis [6], so abrogation of PDIA3 functions could result in decreases in pro-apoptotic pathway activation. In addition to this, PDIA3 interacts directly with STAT3 to suppress pY705 [23]. It is likely that during this interaction, pS727 is inhibited [76]. By reducing the amount of pSTAT3 Y705 in the nucleus, PDIA3 may increase the available pool of cytoplasmic STAT3 capable of being phosphorylated on S727 and, after translocation to the mitochondria, promoting mitochondrial function and cell survival. Alternatively, reduced PDIA3 can function as a ROS scavenger [77], and increased ROS promotes STAT3 phosphorylation on S727 and mitochondrial recruitment [78]. Thus, the ROS increases observed in PDIA3-/- cells could be the mechanistic link between PDIA3 and pSTAT3 S727. Following OGD, we found that pY705 was completely abolished in both WT and PDIA3-/- CMECs, suggesting that Y705 is not important for cell survival in this model. Pharmacological inhibition of STAT3 with stattic resulted in a loss of PDIA3-/- protective effects against OGD. Other studies found that inhibition of STAT3 can be protective against hypoxic injury by blocking translocation to the nucleus [79]. STAT3 has also been found in the ER, where it has a role in regulating calcium homeostasis as key to its anti-apoptotic activities [76]. We observed increased mitochondrial superoxide in PDIA3-/- cells and this may lead to decreased rates of cell growth (Additional file 1: Fig. S1) through downregulation of cyclins involved in cell cycle checkpoints. P21 and P53 are upregulated in response to ROS production, delaying progression from G1 to S phases of the cell cycle [80]. At the same time, it is possible that loss of PDIA3 leads to decreased cell death through increased STAT3 S727 phosphorylation and reduced calcium overload [76]. Further, reduced FAK-S727 STAT3 activation following ER stress in mouse bEnd5 endothelial cells leads to decreased cell survival [33], suggesting an important role for this pathway in preventing apoptosis under injurious conditions. Here, both FAK pY397 and STAT3 pS727 were reduced following OGD, suggesting that partial preservation of this signaling pathway protects these cells. However, the mechanism for how PDIA3-/- increases FAK pY397 under OGD, but not under normoxic conditions requires further investigation.

## Conclusions

**Fig. 7:**
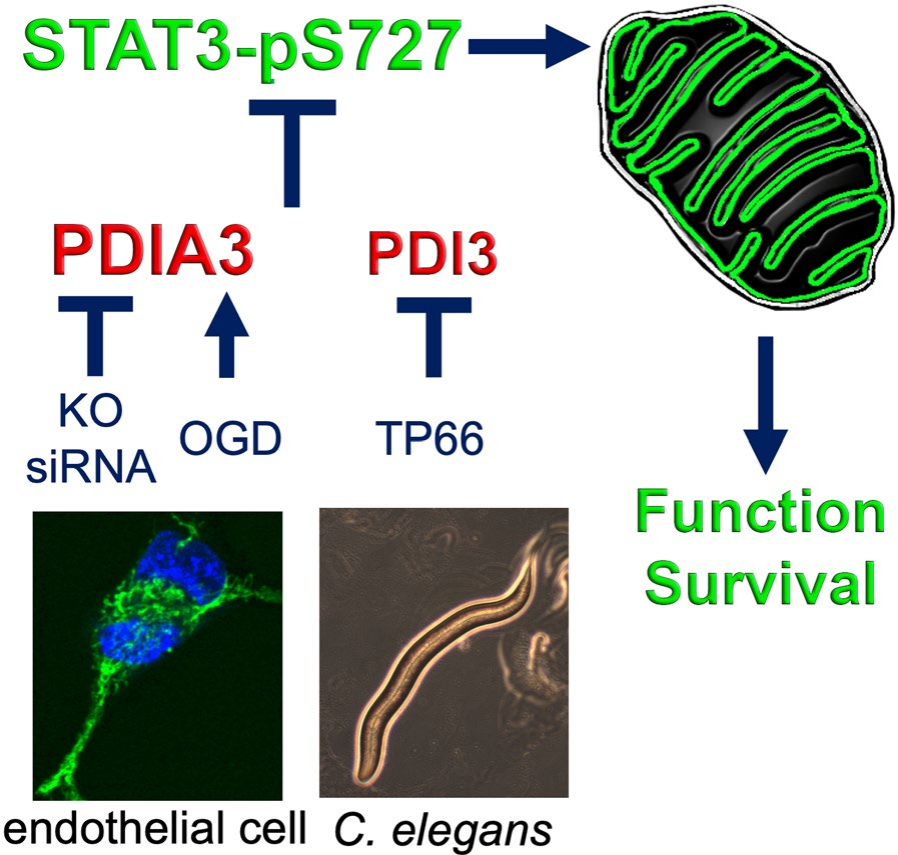
PDIA3 reduces mitochondrial function: Overview. In endothelial cells, PDIA3 inhibits phosphorylation of the S727 residue of STAT3, which promotes mitochondrial bioenergetics. PDIA3 gene knockout with CRISPR or knockdown with siRNA increases STAT3 activation, mitochondrial bioenergetics, cellular function and cell survival under OGD conditions. In *C. elegans*, the PDI-3 homolog plays a similar role in mitochondrial bioenergetics as shown in the TP66 mutant strain

In conclusion, our study defines a novel role for PDIA3 in inhibiting mitochondrial bioenergetics (Fig. 7), whereby decreases in PDIA3 increases respiratory function and reduces cell loss during OGD. This presents an important step forward in understanding PDIA3 functions related to STAT3 signaling and new avenues for therapeutic intervention for diseases such as stroke.

## Supplementary Information

**Additional file 1: Supplemental Figure S1.** PDIA3−/− reduces cellular growth rate in immortalized hCMEC/d3 human endothelial brain cells. **A**) We tested whether PDIA3−/− changed cell replication in CMECs. Counts were performed from brightfield images taken at 24 h intervals (100x magnification). Fields were overlaid with a 225 x 225 μm grid and a total area of 0.4 mm^2^ was counted per field over a total of 3 random fields per well, each field covered 1.47 mm^2^. Cell counts were normalized to cell numbers at 24 h after plating to account for slight differences in plating and cells that did not adhere. **B**) PDIA3−/− and WT cells grew at the same rate over the initial 24–48 h for both genotypes. For WT cells, cell growth continued to increase at 24 h until the cells reached confluence. On the other hand slowed PDIA3−/− growth rate slowed. **C**) We ruled out that cell death was increased in PDIA3−/− which might account for differences in cell number. Lactate dehydrogenase (LDH), released upon cell membrane damage, was not different between WT and PDIA3−/− cells between 48–72 h, a period which showed differing growth rates between the two genotypes.

## Data Availability

All data and analyses of this study have been included in the manuscript. Detailed methods are available upon reasonable request.

## Abbreviations

ER: Endoplasmic reticulum
ERp57: Endoplasmic reticulum protein 57
GRP57: Glucose regulated protein 58
CMEC: Cerebral microvascular endothelial cell
CRISPR: Clustered regularly interspaced short palindromic repeats
FAK: Focal adhesion kinase
FCCP: Carbonyl cyanide 4-(trifluoromethoxy)phenylhydrazone
GAPDH: Glyceraldehyde 3-phosphate dehydrogenase
GFP: Green fluorescent protein
OCR: Oxygen consumption rate
OGD: Oxygen–glucose deprivation
N2: Wild-type *C. elegans* strain
PCR: Polymerase chain reaction
PDHA1: Pyruvate dehydrogenase-A1
PDI-3: Protein disulfide isomerase 3
PDIA3: Protein disulfide isomerase A member 3
pS727: Phosphorylated serine 727
ROS: Reactive oxygen species
S727: Serine 727
siRNA: Short-interfering RNA
STA-1: Signal transducer and activator of transcription 1
STAT3: Signal transducer and activator of transcription 3
TP66: *C. elegans* Strain with a *pdi-3* deletion
WT: Wild-type
Y705: Tyrosine 705
