# Supplementary material for: PDIA3 inhibits mitochondrial respiratory function in brain endothelial cells and C. elegans through STAT3 signaling and decreases survival after OGD
Source: Cell Commun Signal. 2021 Dec 18;19:119. doi: 10.1186/s12964-021-00794-z (PMC8684072; doi:10.1186/s12964-021-00794-z)
Supplement: Supplementary file 1 — Additional file 1: Supplemental Figure S1. PDIA3−/− reduces cellular growth rate in immortalized hCMEC/d3 human endothelial brain cells. A) We tested whether PDIA3−/− changed cell replication in CMECs. Counts were performed from brightfield images taken at 24 h intervals (100x magnification). Fields were overlaid with a 225 x 225 μm grid and a total area of 0.4 mm2 was counted per field over a total of 3 random fields per well, each field covered 1.47 mm2. Cell counts were normalized to cell numbers at 24 h after plating to account for slight differences in plating and cells that did not adhere. B) PDIA3−/− and WT cells grew at the same rate over the initial 24–48 h for both genotypes. For WT cells, cell growth continued to increase at 24 h until the cells reached confluence. On the other hand slowed PDIA3−/− growth rate slowed. C) We ruled out that cell death was increased in PDIA3−/− which might account for differences in cell number. Lactate dehydrogenase (LDH), released upon cell membrane damage, was not different between WT and PDIA3−/− cells between 48–72 h, a period which showed differing growth rates between the two genotypes. [file 12964_2021_794_MOESM1_ESM.pdf]

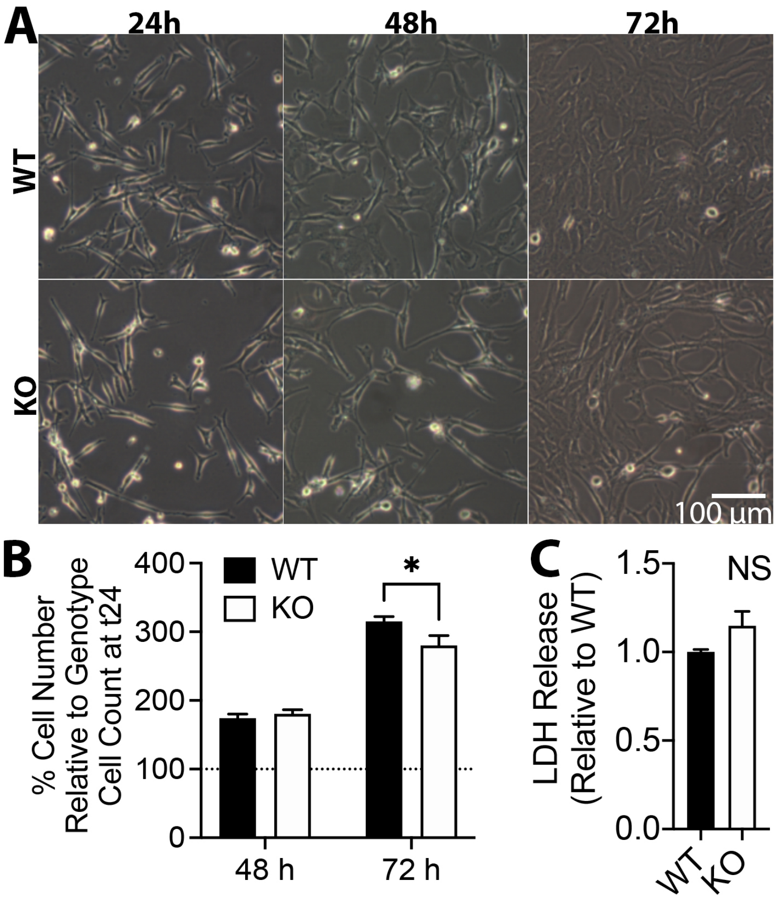

Supplemental Figure S1. **PDIA3-/- reduces cellular growth rate in immortalized hCMEC/d3 human endothelial brain cells**. **A**) We tested whether PDIA3-/- changed cell replication in CMECs. Counts were performed from brightfield images taken at 24 h intervals (100x magnification). Fields were overlaid with a 225 x 225 µm grid and a total area of 0.4 mm^2 was counted per field over a total of 3 random fields per well, each field covered 1.47 mm^2. Cell counts were normalized to cell numbers at 24 h after plating to account for slight differences in plating and cells that did not adhere. **B**) PDIA3-/- and WT cells grew at the same rate over the initial 24-48 h for both genotypes. For WT cells, cell growth continued to increase at 24 h until the cells reached confluence. On the other hand slowed PDIA3-/- growth rate slowed. **C**) We ruled out that cell death was increased in PDIA3-/- which might account for differences in cell number. Lactate dehydrogenase (LDH), released upon cell membrane damage, was not different between WT and PDIA3-/- cells between 48-72 h, a period which showed differing growth rates between the two genotypes.
